# Antioxidant Potential of the Polyherbal Formulation “ImmuPlus”: A Nutritional Supplement for Horses

**DOI:** 10.1155/2014/434239

**Published:** 2014-05-04

**Authors:** Stefano Cecchini, Mariateresa Paciolla, Anna Rocchina Caputo, Alfonso Bavoso

**Affiliations:** ^1^Dipartimento di Scienze, Università degli Studi della Basilicata, Viale dell'Ateneo Lucano 10, 85100 Potenza, Italy; ^2^Consiglio per la Ricerca e la Sperimentazione in Agricoltura-Unità di Ricerca per la Zootecnia Estensiva, 85054 Muro Lucano, Italy

## Abstract

In order to counteract harmful effects of oxidative stress due to pathological conditions or physical exercise, horses are often administered dietary supplements having supposed high antioxidant activities. The aim of the present study was to identify the *in vitro* antioxidant potential of “ImmuPlus”, a polyherbal formulation (Global Herbs LTD, Chichester, West Sussex, Great Britain), containing three medicinal plants (*Withania somnifera, Tinospora cordifolia,* and *Emblica officinalis*), known in Ayurveda for their use in human disease treatment. Extracts obtained by different solvents (water, methanol, ethanol, acetone, and hexane) were tested for total antioxidant capacity, total reducing power, scavenging activity against DPPH radical, and total polyphenol and flavonoid contents. Our results showed that, except as regards hexane, all the used solvents are able to extract compounds having high antioxidant activity, even when compared to ascorbic acid. Regression analysis showed significant correlations between antioxidant properties and polyphenol/flavonoid contents, indicating the latter, known for their beneficial effects on health of human and animal beings, as major components responsible for the strong antioxidant capacities. Moreover, obtained results suggest the effective role of the polyherbal mixture as good source of antioxidants in horses.

## 1. Introduction

Oxygen is an electron acceptor making aerobic organisms capable to use energy supplied with foodstuff. Catabolic processes generate free radicals and other free radical intermediates, also known as reactive oxygen species (ROS), mainly produced in the mitochondrial electron transport chain [1]. Thus, ROS are constantly produced in living beings under normal circumstances. However, other biochemical pathways are responsible for ROS production. During phagocytosis by activated neutrophils, for example, several cellular processes are stimulated including the respiratory burst, whereby the increased oxygen consumption by the cell causes the production of potent oxidant bactericidal substances. Because the activity of ROS released by activated neutrophils is not specific, the process may result in host tissue damage [2].

According to studies carried out in humans, oxidative damages are detrimental in immune function [3]. Moreover, aerobic organisms must possess effective antioxidant defenses that, in part, depend on dietary supply of antioxidants [4] being, by definition, molecules capable of inhibiting the oxidation of other molecules. Thus, antioxidants are able to stop oxidation reactions by removing ROS. Knight [3] showed that an antioxidant supplementation essentially reverses age-associated immune deficiencies, for example, augmenting specific antibody response and decreasing lipid peroxidation. Therefore, dietary antioxidants preserve an adequate function of immune cells against homeostatic disturbances caused by oxidative stress [5] occurring when the ROS generation rate exceeds that of their removal [6]. The excess of ROS is responsible for lipid and protein oxidation, including DNA, as well as peroxidation of unsaturated fatty acids, essential for cellular membrane function [2].

Furthermore, fruits and vegetables are known for their high content in antioxidant nutrients, as well as in vitamin C, vitamin E, and carotenoids, significantly contributing to their total antioxidant capacity [7]. Among compounds of vegetables having this nutraceutical function, polyphenols, secondary plant metabolites, represent the components having the stronger antioxidant capacity [8].

In horse, oxidative stress may be a consequence either of several pathological conditions due to biotic and abiotic factors or of physical exercise [9]. The latter is a potent stimulator of ROS production and it has been indicated as causative agent of oxidative stress in horses [10], even if Kirschvink et al. [11], comparing the blood oxidant status of healthy and heaves-affected horses after physical exercise, showed that the oxidant status is more affected by the air-way disease than by the exercise.

However, both intensive [12] and endurance [13] exercises are able to induce oxidative stress in athletic horses. Robson et al. [14] showed in horses subjected to long-duration exercises a negative impact on the function of the innate immune system that recovers not before three days after race. The observed decrease in respiratory burst activity is probably due to several factors, but it may be ascribed partially to the increase in serum cortisol concentration, as shown immediately after very prolonged exercises [14]. According to the authors, the decreased immune function observed as consequence of long-duration exercises may account for the increase of infectious episodes in horses during athletic training, as shown by Burrell et al. [15]. Moreover, training is able to change the circadian rhythmicity of antioxidant parameters in athletic horses [16], with higher values of plasma antioxidant capacity in trained horses compared to untrained ones.

In order to counteract the harmful effects of oxidative stress, horses are often administered dietary supplements having alleged high antioxidant activities. Among them, herbal extracts seem to have some beneficial properties on health status of horses [17–19].

This current study was undertaken to evaluate the antioxidant potential of a polyherbal formulation by using different standard tests. This herbal mixture, marketed under the name of “ImmuPlus” by Global Herbs LTD (Chichester, West Sussex, Great Britain), is suggested as nutritional supplement for the function of immune system of horse and contains three powdered herbs in equal proportions:* Withania somnifera* (ashwagandha),* Tinospora cordifolia* (guduchi), and* Emblica officinalis* (amlaki). These herbs are known in Ayurveda, the Indian traditional health care system approved for its efficacy from the World Health Organization, for their immunostimulant efficacy for animal and human beings [20].

## 2. Materials and Methods

### 2.1. Extract Preparation

Extracts of “ImmuPlus” were prepared by soaking 40 g of dry powdered herbal mixture in 200 mL of different solvents (water, methanol, ethanol, acetone, and hexane) at room temperature (20°C) for 48 h under gentle shaking. The resultant extracts were centrifuged at 3000 ×g for 20 min at 4°C. The supernatants were then collected and filtered progressively up to 0.45 *μ*m before concentrating them using a lyophilizator (water extract) and a rotary evaporator with the water bath set at 40°C. The percentage yield of extracts is reported in Table 1. Each extract was suspended in the same solvent of extraction at the concentration of 1 mg mL^−1^ before phytochemical analyses.

### 2.2. Total Antioxidant Capacity

Total antioxidant capacity (TAC) of extracts was determined using the ferric ion reducing antioxidant power (FRAP) assay as indicated by Benzie and Strain [21]. TAC of sample was derived from a standard curve of iron (II) sulfate heptahydrate at concentrations ranging from 62.5 to 1000 *μ*M (Pearson's correlation coefficient: *r* ^2^ = 1) and it was expressed as *μ*g iron sulfate equivalents (ISE) mg^−1^ dry weight (dw) of extract. Aqueous ascorbic acid (AA) solutions (1 mg mL^−1^) were used as positive controls for TAC.

### 2.3. Total Reducing Power

Total reducing power (TRP) of extracts was determined according to the method of Oyaizu [22]. TRP of sample was derived from a standard curve of AA ranging from 4.69 to 300 *μ*g mL^−1^ (Pearson's correlation coefficient: *r* ^2^ = 0.99) and it was expressed as *μ*g AA equivalents (AAE) mg^−1^ dw of extract.

### 2.4. Free Radical Scavenging Activity

Free radical scavenging activity (FRSA) of extracts was determined by the 1,1-diphenyl-2-picrylhydrazyl (DPPH) free radical scavenging assay using the modified Blois' method [23], in which samples are mixed with a 0.5 mM DPPH solution and kept for 20 min in darkness before absorbance reading. The percentage of DPPH scavenging was calculated by measuring the absorbance of the extract and applying the following equation:

(1) $$\begin{array}{c} \%\;\text{of}\;\text{inhibition}=\left[1-\left(\frac{A_{\text{s}}}{A_{0}}\right)\right]\times 100, \end{array}$$

where *A*_s_ is the absorbance of sample and *A*_0_ is the absorbance of the DPPH solution. Aqueous AA solutions (1 mg mL^−1^) were used as positive controls for FRSA.

### 2.5. Total Polyphenol Content

Total polyphenol content (TPC) was determined using the Folin-Ciocalteu method, as described by the International Organization for Standardization (ISO) 14502-1 [24]. TPC in samples was derived from a standard curve of gallic acid ranging from 10 to 50 *μ*g mL^−1^ (Pearson's correlation coefficient: *r* ^2^ = 0.99) and it was expressed as *μ*g gallic acid equivalents (GAE) mg^−1^ dw of extract.

### 2.6. Total Flavonoid Content

Total flavonoid content (TFC) was determined with aluminum chloride (AlCl_3_) according to Zhishen et al. [25] using quercetin as a standard. TFC was calculated from a quercetin standard curve ranging from 12.5 to 400 *μ*g mL^−1^ (Pearson's correlation coefficient: *r* ^2^ = 0.99) and it was expressed as *μ*g quercetin equivalents (QE) mg^−1^ dw of extract.

### 2.7. Statistical Analysis

Analytical data, presented as mean and standard deviation, are the average of three separate analyses each one performed in triplicate. One-way analysis of variance (ANOVA), performed using SigmaPlot for Windows Version 11.0 statistical software, was used for determining the influence of different solvents on evaluated phytochemical characteristics. Differences between means were determined by Tukey's pairwise comparisons and a probability level of *P* ≤ 0.05 was considered significant. Linear regression analyses were performed in order to verify correlations between antioxidant parameters and total polyphenol and flavonoid contents.

## 3. Results

In this study, the antioxidant properties and the total polyphenol and flavonoid contents of different extracts of the polyherbal formulation “ImmuPlus,” a horse dietary supplement, have been investigated. As shown in Table 2, results revealed that antioxidant properties, analyzed as TAC, TRP, and FRSA, and polyphenol and flavonoid contents in the ethanol extract were higher when compared to aqueous, methanol, acetone, and hexane extracts, even if the percentage yield of extracts is quite different being higher when water was used as solvent (Table 1). Specifically, as regards hexane, its extracts have shown very low antioxidant properties and no analyzable amounts of polyphenols and flavonoids. According to our results, TAC of extracts was not less than one-third of TAC values obtained by AA, being 7989 ± 597 *μ*g ISE mg^−1^, if hexane extract values are excluded from comparison. Even further, the same can be said with regard to FRSA, in which aqueous, methanol, ethanol, and acetone extracts have about the fifty percent of the observed AA scavenging activity. At the same time, TRP analysis, in which data are expressed as AA equivalents, has revealed that the reducing power of different extracts was between 13% and 25% of the reducing power of pure AA. TPC varied considerably from 150.01 (±8.38) *μ*g GAE mg^−1^ of aqueous extracts to 236.14 (±18.74) of ethanol extracts; at the same time, higher values of TFC were found in ethanol extracts (215.61 ± 5.38 *μ*g QE mg^−1^).

Correlations among antioxidant properties and polyphenol and flavonoid contents are shown in Table 3. The correlation matrix showed a significant linear correlation among all the five analytical methods (*P* ≤ 0.001), proving that all the methods are effective indicators of antioxidant properties of the polyherbal formulation.

## 4. Discussion

In living systems, the constant ROS generation causes tissue and molecule damage that may lead to various diseases. Thus, the use of adequate amounts of antioxidants as dietary supplements in human and animal beings seems appropriate for their recognized capacity to counteract ROS, mainly when they are exposed to unfavorable and stressful conditions. Besides, reduced dietary intake of antioxidants has been related to a decrease in antioxidant defense and to an increased susceptibility to oxidative stress [26]. As regards horses, several pathologies and physical exercise have been identified as the causes of oxidative stress [2], suggesting that an antioxidant supplementation can be useful to support the health status, although some dietary supplements are used without their efficacy having been tested before.

Many studies have been performed in order to identify nutraceutical substances in herbs or in herbal mixture of traditional medicine. Plants from Ayurveda have several antioxidant compounds (tannic acid, polyphenols, flavonoids, tocopherol, carotenoids, ascorbate, etc.) acting probably in a synergistic way [27].

According to O'Neill et al. [18], oral treatment with* Echinacea angustifolia* extracts in healthy horses increases the phagocytic activity, boosts peripheral lymphocyte counts, and stimulates neutrophil migration, as well as increases peripheral red blood cells and hemoglobin concentration. On the contrary, single doses of extracts from black tea, orange peel, cranberry, and ginger do not seem able to influence the oxidative stress and the antioxidant status in intensely exercising horses, even if, according to the authors in [28], long-term supplementation would be necessary to investigate whether these extracts may reduce the oxidative stress.

Regarding the herbs composing the analyzed polyherbal mixture “ImmuPlus,” early studies showed that they possess antioxidant activities and have potential as therapeutic agents to modulate the immune system of human and animal beings [20, 29, 30]. These three herbs have an adaptogenic potential and, according to the Ayurvedic pharmacology, they are classified in the clinical speciality Rasayana [31], whose purpose is to restore spirit and vitality and thereby attain longevity. In particular,* E*.* officinalis* contains two hydrolysable tannins, emblicanin A and B, with low molecular weight having a very strong antioxidant action [32], whereas* W*.* somnifera* contains, among polyphenol substances, high amounts of the flavonoid catechin, having strong antioxidant properties [33]. As regards* T*.* cordifolia*, Premanath and Lakshmidevi [34] have shown, besides radical scavenging activity, strong lipid peroxidation inhibitory activity of ethanol leave extracts, which justifies the ethnomedical use of this plant.

The correlations shown by the present study among antioxidant properties and polyphenol and flavonoid contents indicate not only that all the five methods are reliable indicators of antioxidant activities of the polyherbal mixture, but also that the antioxidant properties are mainly due to its polyphenol content and, among polyphenols, its flavonoid content, as well. Although a correlation between phenolic content and antioxidant activity was not always demonstrated [35], our results are in agreement with others' findings obtained in other plant materials [36, 37].

Even further, the high TRP values of extracts show that the polyherbal mixture contains substances that are electron donors able to reduce the lipid peroxidation processes [38]. The strong FRSA of the polyherbal mixture extracts are dependent on phenolic compounds. In fact, phenolic substances are considered very important herbal constituents as their hydroxyl groups bestow the scavenging activity.

## 5. Conclusion

Our attempt to scientifically demonstrate possible advantages by the use of the polyherbal formulation “ImmuPlus” as equine nutritional supplement has shown its high antioxidant potential, demonstrated by different analytical methods, with results comparable to those of AA used as standard compound. The* in vitro* study indicates that this polyherbal supplement is a significant source of natural antioxidants which could be helpful in preventing harmful damage by oxidative stress. The strong correlations between antioxidant properties and TPC/TFC in extracts show that polyphenols and flavonoids are major components which are principally responsible for the high antioxidant capacities of the polyherbal formulation. Both of these classes of substances are known for their beneficial effects on health of human and animal beings.

It is believed that in order to validate our results further investigations are needed to clarify the* in vivo* potential of this polyherbal mixture in alleviating negative effects of oxidative stress induced by several pathologies and by physical exercise in horses.

## Figures and Tables

**Table 1:** Percentage yield of extracts obtained with different solvents of the polyherbal formulation “ImmuPlus.”

Water	Methanol	Ethanol	Acetone	Hexane
18.15 ± 1.75^a^	6.93 ± 0.87^b^	2.25 ± 0.51^c^	2.00 ± 0.23^c^	0.39 ± 0.10^d^

Values with different letters are statistically different (*P* ≤ 0.05).

**Table 2:** Analysis of antioxidant properties and polyphenol and flavonoid contents of different extracts of the polyherbal formulation “ImmuPlus.”

Solvent	TAC	TRP	FRSA	TPC	TFC
Water	1375 ± 150^a^	171 ± 16^a^	24.4 ± 3.5^a^	150 ± 8^a^	85 ± 15^a^
Methanol	1383 ± 217^a^	129 ± 14^a^	18.6 ± 4.2^a^	159 ± 9^a^	132 ± 2^b^
Ethanol	2538 ± 248^b^	255 ± 25^b^	35.3 ± 2.1^b^	236 ± 19^b^	216 ± 5^c^
Acetone	1406 ± 150^a^	130 ± 9^a^	19.1 ± 3.6^a^	162 ± 20^a^	147 ± 6^b^
Hexane	333 ± 32^c^	9 ± 4^c^	0^c^	0^c^	0^d^

In each column, values with different letters are statistically different (*P* ≤ 0.05).

TAC: total antioxidant activity (*μ*g ISE mg^−1^), TRP: total reducing power (*μ*g AAE mg^−1^), FRSA: free radical scavenging activity (% of DPPH inhibition), TPC: total polyphenol content (*μ*g GAE mg^−1^), and TFC: total flavonoid content (*μ*g QE mg^−1^).

**Table 3:** Correlation matrix among antioxidant properties and polyphenol and flavonoid contents of the polyherbal formulation “ImmuPlus.”

	TAC	TRP	FRSA	TPC	TFC
TAC	1	0.980*	0.951*	0.970*	0.945*
TRP	0.980*	1	0.977*	0.971*	0.912*
FRSA	0.951*	0.977*	1	0.947*	0.913*
TPC	0.970*	0.971*	0.947*	1	0.949*
TFC	0.945*	0.912*	0.913*	0.949*	1

When the asterisk appears, the correlation is significant (*P* ≤ 0.001).
